# Randomised evaluation of the Italian medicines use review provided by community pharmacists using asthma as a model (RE I-MUR)

**DOI:** 10.1186/s12913-015-0791-6

**Published:** 2015-04-21

**Authors:** Andrea Manfrin, Trudy Thomas, Janet Krska

**Affiliations:** Medway School of Pharmacy, University of Kent and Greenwich, Central Avenue Chatham Maritime, Chatham, ME4 4TB Kent UK

**Keywords:** Asthma, Medicines use review, Cluster randomised controlled trial (RCT), Community pharmacy

## Abstract

**Background:**

The Italian Ministry of Health decided to introduce community professional services in 2010. This trial provides an opportunity to evaluate the outcomes of a new professional pharmacy service: Italian Medicines Use Review (I-MUR) aimed at reducing the severity of asthma and its associated costs.

**Methods/Design:**

This is a cluster randomised controlled trial of the I-MUR service. Data will be collected over time before, during and after pharmacists’ intervention. Fifteen Italian regions will be involved and it is aimed to recruit 360 community pharmacists and 1800 patients. Each pharmacist will receive training in medicines use review, recruit five patients, administer the Asthma Control Test and provide the I-MUR service. Pharmacists will be allocated to different groups, one group will be trained in and provide the I-MUR service immediately after completion of the baseline ACT score, the other group will receive training in the I-MUR and provide this service three months later. Group allocation will be random, after stratification by region of Italy. The I-MUR service will involve gathering data following each patient consultation including demographic details, patients regular medications, including those used for asthma, their attitude towards their medications and self-reported adherence to treatments. In addition, pharmacists will identify and record pharmaceutical care issues and any advice given to patients during the I-MUR, or recommendations given to doctors. Pharmacists will upload trial data onto a web platform for analysis. The primary outcome measure is the severity of asthma before, during and after the I-MUR assessed using the Asthma Control Test score. Secondary measures: number of all active ingredients used by patients during and after the I-MUR, number of pharmaceutical care issues identified during the I-MUR, patients’ self-reported adherence to asthma medication during and after the I-MUR, healthcare costs based on the severity of asthma, before, during and after the I-MUR service provision.

**Discussion:**

This study has been developed because of the need for a new way of working for pharmacists and pharmacies; it is the first trial of any community pharmacy-based pharmaceutical care intervention in Italy. The results will inform future policy and practice in Italian community pharmacy.

**Trial registration number:**

ISRCTN72438848.

## Background

### What is the problem to be addressed?

It is estimated that around 300 million people in the world have asthma with an additional 100 million cases predicted by 2025 [1]. The number of disability-adjusted life years (DALYs) lost to asthma worldwide has been estimated to be around 15 million per year with an estimated one in every 250 deaths worldwide caused by asthma [1].

Prevalence of asthma in the European adult population, age 18–44, varies from country to country, with the highest value in Sweden (20.62%) and the lowest in Bulgaria and Lithuania (2%); in Italy it is 6.2% [2].

The total cost of respiratory diseases in the 28 countries of the EU alone amounts to more than €380 billion annually with asthma accounting for €33.9 billion [3]_._ The total bill includes the costs of direct primary and hospital healthcare (around €55 billion), the cost of loss of production (nearly €42 billion) and the monetized DALYs lost (at least €280 billion).

Overall cost of asthma, including individual direct costs, indirect costs and intangible quality of life costs have been related to severity of asthma [4-6].

In 2000, a COI (Cost of Illness) study was carried out in Italy in the frame of the Italian Study on Asthma in Young Adults. The mean annual costs for an asthmatic patient was €741 (95% CI: 599–884) ranging from €379 (95% CI: 216–541) for well-controlled asthmatics to €1,341 (95% CI: 978–1,706) for poorly controlled cases that accounted for 46.2% of the total cost [7].

### Pharmacists’ role in asthma management

Pharmacists have the potential to improve asthma severity through interventions, which include counseling on inhaler technique, medicine therapy management and medicines use review (MUR) [8-12].

A portrait of the pharmacy profession around the world suggests a wide variation in the practice of pharmacy, not only between countries but also within countries [13].

In England, the Isle of Wight Respiratory project, involved health care practitioners including community pharmacists. The aim was to reduce respiratory death, hospital admission and the cost of respiratory medication. In this study, general practitioners referred patients to their community pharmacist for inhaler technique training. The results showed that reliever therapy costs dropped by 22.7%, emergency admissions due to asthma reduced by 50%, deaths fell by 75% and hospital inpatient costs fell by 66%. A review on the NICE shared database highlighted that the training of health care practitioners carried a cost, which was paid back seven times over with the reduction in cost of bronchodilators within one year of the project [14].

In Denmark, a prospective controlled multicenter study, aimed to improve asthma severity (status) by increasing the participation of pharmacists and promoting cooperation with patients and the patient’s general practitioner (GP). The study recruited 500 asthmatic patients from primary care, and found improvements in asthma symptoms status, quality of life and days of sickness [15].

In Finland, a 10-year asthma program was undertaken from 1994 to 2004 to improve asthma care and prevent asthma costs increasing, using general practitioners, nurses, and pharmacists. The main goal of this program was to lessen the burden of asthma to individual and to society. Pharmacists provided patients with written or oral information on preventers and relievers during 98% of asthma medication purchases, plus gave instruction on inhalation technique to 98% of newly diagnosed asthmatic patients and 34% of patients with existing disease. Pharmacists’ intervention combined with the ones provided by GPs and nurses contributed to reduce the number of days patients were in hospital which fell by 54%; the increasing cost of asthma had stopped and the annual cost per patient decreased by 36% from €1,611 to €1,031 [16].

An Australian multi-site randomised controlled trial compared pharmacist counseling on asthma, asthma medication and associated lifestyle advice with a control group providing usual care. Fifty pharmacies were enrolled and randomly allocated to one of the two groups (randomisation conducted at pharmacy level), the intervention group with 191 patients and the control group with 205 patients. In both groups questionnaires and spirometric testing were carried out at baseline and at six months. The main outcome measure was asthma severity/control status assessed by a tool adapted from the National Asthma Council. The percentage of patients in the intervention group classified as having severe asthma declined from 87.9% to 52.7% (p < 0.01), whilst the change in the control group the change was not significant (71.2% to 67.9%; p = 0.11). These authors used a multilevel logistic regression model to adjust for the difference in asthma severity at baseline and to account for any possible effect from clustering. Both pharmacist groups administered questionnaires and spirometric testing at baseline and six months later; it was found that patients in the intervention group were almost three times more likely to change from the severe asthma category to the not severe category than patients in the control group (OR 2.68, 95% CI 1.64 to 4.37 p < 0.01) [17].

### Medicines use reviews in asthma

The Medicines Use Review (MUR) service was first introduced in England in 2005 as an advanced service in the community pharmacy contractual framework. The aim of the MUR is to achieve a concordant consultation about medicine-taking by establishing the patient’s actual use, understanding and experience of taking their medicines, with the ultimate aim of improving the clinical and cost-effectiveness of prescribed medicines, reducing medicines wastage and improving patient outcomes through improved adherence [18].

Portlock et al. [19] conducted a study in England in which 965 MURs were undertaken in patients with asthma. Pharmacists identified that 37% of the patient population were primarily non-adherent (i.e. collected < 75% of intended asthma prescriptions in the previous 12 months) and a further 31% had secondary adherence issues (i.e. not taking their medications in the way they had been intended). Pharmacists made 1,787 interventions (mean 1.8 per MUR consultation) of which 41% were device checks, 10% were referral to a GP or nurse and 49% were educational.

Another study involving 154 patients with asthma receiving an MUR found that the proportion of patients whose asthma was not controlled fell from 59% to 45% (p < 0.01), 30% of patients were referred to their GPs or asthma nurse as a result of their MUR and, of those referred, 70% had a treatment or dosage change [20].

### Italian medicines use review (I-MUR)

This study is about developing a similar service in Italy where no such services exist. The I-MUR has been designed as a structured interview, supported by a quantitative questionnaire enabling pharmacists to capture in a systematic way a patient’s demographic data, quantity and type of medicines used, their knowledge about the medicines used, their adherence to the medicines, actual complaints (e.g. shortness of breath, chest tightness, night time waking, need for rescue medicines, limitation on activity including exercise), plus any pharmaceutical care issues identified by the pharmacists, the advice given to physicians and to patients, including healthy living advice, using an online platform (Qualtrics®).

The Italian government is considering introducing the I-MUR service, but at present there is no evidence from Italy on the feasibility, acceptability or effectiveness of such a model. Hence there is a need to gather robust data on all aspects of the potential new service to inform service design and delivery.

A pilot study was carried out between October 2012 and January 2013, to assess the feasibility of Italian community pharmacists delivering I-MURs to patients with asthma [21]. In this phase, the MUR template used in England was adapted and supplemented with a structured interview to be conducted with patients. The template also included the classification of pharmaceutical care issues (PCIs) which were potentially identifiable by pharmacists during the patient consultation. Pharmaceutical care issues were classified using the method developed by Krska et al. [22] where they are defined as “an element of a pharmaceutical need which is addressed by pharmacists”. This enabled pharmacists to categorize, using a systematic approach, all issues they found during the consultation.

This pilot study was carried out in four Italian regions and involved 74 pharmacists who provided the I-MUR to 895 patients during a four-month period, following training [23]. The training provided was evaluated and pharmacists’ views on the service provision sought through focus groups. Poor adherence was found in 45% of the 895 patients and only 18% had either no asthma-related problems – either actual complaints or medicine-related problems. Pharmacists identified pharmaceutical care issues in 60% of patients; they provided 1008 items of medicine-related advice to GPs and 1321 to patients, plus 1219 items of healthy living advice. Pharmacists’ clinical knowledge increased by 24%; following training and I-MUR service provision.

Following this pilot study, which focused on pharmacists, a second study, conducted between October and November 2013, sought to obtain patients’ feedback and GPs’ views on the I-MUR service provision. All patients who had received the I-MUR service previously were invited to complete a feedback questionnaire. Responses were obtained from 246 patients (27% response rate). The questionnaire found that 50% of patients were neither worried nor had problems with their medicines before having the I-MUR, but 75% of them confirmed that they benefited from the service. Seventy-five percent of patients also indicated they felt involved in all the discussion, 37% confirmed that I-MUR found problems with their medications and 27% agreed that changes were made to their medication after the I-MUR. Half the patients would consider having another I-MUR and 85% would recommend the service to other patients [24]. A focus group was held which was attended by four GPs. GPs’ views about I-MUR was positive. They identified the potential for the I-MUR to improve patients’ adherence, quality of life, result in safer use of medicines and better health care outcomes. GPs suggested developing and sharing training sessions, underlining the fact that GPs and pharmacists should work together, empowering each other, and sending a consistent message to their patients [25].

In Italy no other work has been conducted on asthma involving community pharmacists, therefore this current study is needed to determine whether the I-MUR can contribute to improve asthma outcomes and to demonstrate whether the service provides clinical and cost benefits, as these were not included in pilot work. The study will provide evidence about the practicability and costs of provision and the effectiveness of the I-MUR in asthmatic patients.

## Methods/Design

### What are the principal research questions to be addressed?

Is the I-MUR service provided by community pharmacists in Italy effective in:

I. Reducing the severity of asthma as assessed by the Asthma Control Test (ACT) score?
II. Optimising the number of medicines (active ingredients) used by patients?
III. Identifying and resolving pharmaceutical care issues?
IV. Improving patients’ adherence to asthma medications?
V. Reducing costs (direct and indirect) related to the reduction of the severity of asthma?

### Risks and safety issues

During this study there will be no risks for patients. Those patients receiving the I-MUR service will be at no greater risk than those receiving usual clinical care. Pharmacists will ask their patient to complete the ACT tool and will provide the I-MUR service. They will not dispense or administer any medications based on the results of the ACT or I-MUR results. Pharmacists will neither be involved in the interpretation of diagnostic tests, nor their results.

The activity involved in this study will not expose pharmacists to any risk beyond their normal professional practice.

### Study design

This will be a clustered randomised controlled trial with multiple data collection points.

Data will be collected from 360 Italian pharmacists who will provide I-MUR services to 1800 patients over a nine-month period.

A standard randomised study design would mean that some pharmacists would not provide the I-MUR service to their patients, in order to form a control group, an approach which could lead to demoralise and disengage pharmacists who will not have the chance to provide the service. In addition, individual pharmacists may differ in their practices, and their patients may be cared for by GPs with different practices. Hence the study design must minimise the impact of any individual pharmacist or GP on the management of asthma. To avoid these problems, it was decided to design a cluster randomised control trial in which pharmacists are randomly allocated to provide the intervention to their patients either early or later during the study period. Randomisation will be at pharmacist level, not at patient level, with each pharmacist representing a cluster and outcome being measured for individuals within those clusters [26].

Data will be collected before, during and after intervention delivery, to assess if timing of the intervention affects the primary outcome: the severity of asthma.

### Study setting

Italy is a country with nearly sixty million people and it is divided in 20 regions. Pharmacist participants will be selected from different regions throughout the country to reduce potential impact from North–South climatic variation, which could impact asthma severity. Selected regions include Trentino Alto Adige, Lombardia, Sicilia, Puglia, Sardegna, Piemonte, Valle d’Aosta, Veneto, Friuli Venezia Giulia, Toscana, Emilia Romagna, Marche, Abruzzo, Lazio and Campania. Pharmacists will be stratified by region before randomisation. These geographical areas were identified and selected by the Italian Pharmacists’ Federation following attendance at regional presentations of the study conducted by the principal investigator (PI).

### What is the aim and what are the planned trial interventions?

#### Aim

To evaluate the outcomes of I-MUR interventions provided by Italian community pharmacists in an asthmatic patient population.

#### Planned intervention

Pharmacists will provide the I-MUR service following administration of the ACT at either T0 or T3 depending on group allocation, recording details of all PCIs identified and advice provided during the I-MUR consultation.

The I-MUR intervention covers the following areas: patients’ demographics, their regular medications (active ingredients) including those used for asthma, patients’ attitude towards their medications and adherence to treatments assessed with the I-MUR interview, pharmaceutical care issues identified by the pharmacist, pharmacist’s advice given to patients, including healthy living advice, pharmacist’s advice given to Doctors, pharmacist’s own view on the potential benefit to the patient of the MUR service they provided.

Following each consultation, pharmacists will be required to enter the results into the web-based template provided, maintaining patient anonymity.

### Sample selection

#### Pharmacists

##### Who wrote to the pharmacists?

An invitation letter and a summary of the study was prepared by the PI and sent to FOFI.

FOFI distributed the above information to all community pharmacists in all the identified regions inviting pharmacists to participate in the study.

##### How did pharmacists consent?

Pharmacists who expressed interest in participating were invited to attend a regional presentation. At the regional presentations, which took place between March and April 2014, the PI outlined the study protocol and the enrollment criteria and participants were given a detailed participant information sheet.

The regional team leader collated names of pharmacists who expressed interest over the next three to four weeks and the first 36 who met the inclusion criteria (see below) were selected for study inclusion and invited to the I-MUR training.

### Pharmacist inclusion and exclusion criteria

Pharmacies must have

- a private area for private consultation with patients;
- an internet connection available where the consultation will take place.

Pharmacists must

- be qualified and registered with the Italian Pharmacy Board practising in Italy;
- have at least one year of experience in providing advice to patients;
- already provide one or more services such as blood pressure monitoring, smoking cessation, cholesterol monitoring, signposting, food intolerance testing., in order to demonstrate advanced consultation skills and experience;
- be able to attend training sessions.

Pharmacies must be excluded if they

- have no internet access; or
- no consultation room.
- are currently involved in any other clinical pharmacy research project.

### Patient selection

#### Patient inclusion and exclusion criteria

Patients will be identified by the individual pharmacists from their medication history, from their prescriptions, or by referral from GP, according to inclusion and exclusion criteria listed below.

Patients must:

- be at least 18 years of age;
- have been diagnosed with asthma, for at least six months before enrolment to the study;
- have a prescription(s) for asthma medication with R03 as ATC code (Anatomical, Therapeutic Chemical Classification), or drugs for obstructive airways disease.

Patients must be excluded if they

- have terminal illness (defined as an advanced stage of a disease with an unfavourable prognosis and no known cure) as identified by the pharmacists through the prescription coding;
- are currently enrolled in another clinical trial;
- do not self-administer their inhaler;
- are not able to communicate well in Italian both written and spoken.

### Recruitment and informed consent

Pharmacists, after having assessed patients’ eligibility for the study, will provide an information letter and consent form to each patient, who will be given an opportunity to consider their participation. All signed consent forms will be retained by the pharmacist in the pharmacy.

### Procedures

#### Pharmacist randomisation and blinding

**Figure 1:**
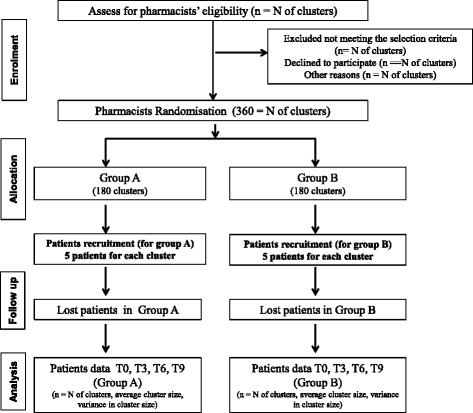
Flow diagram for the study (Adapted from BMJ 2012; 345:e5661 doi:10.1136/Bmj.e5661, September 2012).

An academic member of staff at Medway School of Pharmacy, experienced in clinical trials will be in charge of the randomisation process. Pharmacists will be randomly allocated to group A or B. Pharmacists will be stratified by location, prior to randomisation, using computer-generated random numbers. Blinding will not be possible either at pharmacist or patient level because the nature of the intervention requires their full knowledge. However the PI, as assessor of the main outcome measure, severity of asthma, will remain blind throughout. As group allocation will be intrinsic to the data gathered for each patient, in order to maintain blindness, the PI will access the data only after all patients have been followed up at three months (see Figure 1).

### Pharmacist training

Training sessions will last for a half day and will be provided in each region by the PI, a fully trained and qualified pharmacist accredited to provide MURs in England since 2005. The training supports the development of pharmacists’ clinical skills and knowledge using question and answer sessions, mock interviews and role-play. The training material was developed and evaluated during earlier work. Contributions to the training will also be included from local respiratory consultants and general practitioners who will cover asthma management, physiopathology, medical conditions, different treatments according to asthma severity and GINA guidelines. Pharmacists from Groups A and B will be trained at different times. The use of the ACT test will be explained to both groups before T0 but only group A will receive the I-MUR training at this time point; group B will receive this training just before T3.

### Study timeline

The planned duration of the study is nine months.

### ACT

The ACT (primary outcome measure) is a patient-completed questionnaire with five items assessing asthma symptoms (daytime and nocturnal), use of rescue medications, and the effect of asthma on daily functioning. Each item includes response options corresponding to a 5-point Likert scale, which are summed to yield a score ranging from 5 (poor asthma control) to 25 (complete asthma control). Schatz et al. [27] found that a cut off score of 19 or less identifies patients with poorly controlled asthma.

#### How will the ACT test be administered?

Pharmacists will administer ACT tests to their patients in the pharmacy consultation area allowing patients’ privacy to read the questions and take the necessary time to respond.

### I-MUR

This will take place in the pharmacy private consultation area. The estimated time for the consultation will be 26 minutes based on phase one experience.

### Data collection

Data collected during the I-MUR will include patient demographics, their regular medications (active ingredients) including those used for asthma, patients’ attitude towards their medications and adherence to treatments assessed during the I-MUR interview, PCIs identified by the pharmacist, pharmacist’s advice given to patients, including healthy living advice, pharmacist’s advice given to doctors, pharmacist’s own view on the potential benefit to the patient of the MUR service they provided.

Following each consultation, pharmacists will be required to enter the data from each consultation into the web-based template provided, maintaining patient anonymity. This process was developed and tested during earlier work [21].

**Figure 2:**
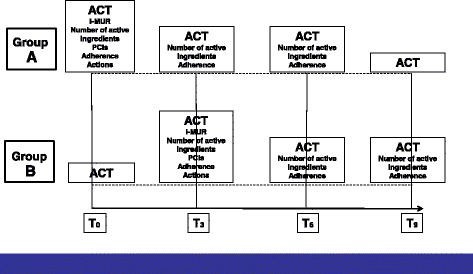
Study timeline, showing activities provided by pharmacists and information gathered.

Data collected at multiple time points will be patients’ responses to ACT, adherence to asthma treatment and number of active ingredients (see Figure 2).

### Proposed sample size

It is aimed to obtain a final sample size of 360 pharmacists and 1800 patients, requiring each pharmacist to provide the intervention to five patients. The power calculation for this study has been conducted taking into consideration the intra-cluster and inter-cluster correlation (ICC) using SPSS sample power calculation. The ICC represents the correlation between responses of individuals in the same clusters which is a proportion of the total variation explained by variation between clusters [28] and its value ranges between 0 and 1. For this study it was decided to use an ICC = 0.02 as this was used in a similar study conducted in Australia [17]. This is based on an estimated 20% difference in asthma severity (ACT score), which may be achieved, again based on the Australian study [17], which found that the proportion of patients classified as having severe asthma declined from 87.9% to 52.7% following pharmacist intervention. This is based on an anticipated 40% improvement in asthma severity following pharmacist intervention; the difference will be measured by comparing the ACT score gathered at baseline to T3, T6, T9 for each patient, where an ACT score ≤ 19 means not well controlled asthma and an ACT score ≥ 20 well controlled asthma.

Four options were considered (Table 1) all using d = 20% and alpha = 0.05, but considering different numbers of pharmacists and patients per pharmacy. Option d was chosen, as it has a power of 95%. The calculated ratio between pharmacists and patient in order to achieve the above results is 1:4. The attrition rate was estimated as 23% based on the Australian study, which had an attrition rate of 14%, but following which the authors suggested that this percentage could be in the range of 14-25% [17]. Therefore pharmacists will be required to recruit five patients instead of four. Based on an attrition rate of 23%, 396 patients will not complete the study, leaving 1386, which is still sufficient to attain option d (693 patients per group).

**Table 1:** **Elements for the evaluation of the power analysis**

**Options**	**Power level**	**ICC**	**d**	**Alpha**	**Number of pharmacists per group**	**Number of patients per group**	**Pharmacists patients ratio**
A	90%	0.02	20%	0.05	115	575	1:5
B	90%	0.02	20%	0.05	140	560	1:4
C	95%	0.02	20%	0.05	140	700	1:5
D	95%	0.02	20%	0.05	173	693	1:4

### Data analysis

In studies where the important baseline factors seem well balanced, it is likely that any differences in outcome between intervention and control groups are a real effect of treatment, which is one component of the internal validity. Patients’ location, age, gender and ACT score will be collected and compared at baseline. This baseline ACT score will provide information regarding the level/severity of asthma prior to intervention delivery.

Quantitative data gathered through the web platform (Qualtrics^©^) will be exported and analysed using SPSS version 22. Patient location, age, gender and ACT score will be compared at baseline.

Asthma severity assessed by ACT score will be analysed over time within individual patients to obtain a percentage change score. Scores will be compared between groups at all times points. The number of active ingredients in use and reported adherence to treatment will be compared at three time points after provision of the I-MUR. The number of PCIs identified and advice given by pharmacists will be captured for both groups separately and combined. Any change in the severity of asthma and the accompanying percentage variation in cost (based on peer reviewed published data) will also be assessed. It will be possible to compare data before and after the I-MUR intervention for group A and B, separately and combined. In addition findings will be analysed by region.

A significance level (alpha) of 0.05 will be used in all analyses. Between-group differences at baseline will be compared using independent Student’s t-test for continuous parameters and Pearson’s χ^2^ will be used for categorical parameters. Outcomes for continuous parameters will be evaluated using repeated measures multivariate ANOVA.

The analysis will be conducted in two steps. Step one will represent an interim analysis after the first six months, after completion of T6, by which time all patients will have been followed up for three months after the intervention. Step two will represent the final analysis, which will be conducted at the end of the study after T9.

### Primary outcome measure

#### Primary outcome

- Severity of asthma: before, during and after the I-MUR service provision assessed using the ACT score.

#### Secondary outcomes

- Number of active ingredients used by patients during and after the I-MUR service provision, as reported by patients.
- Number of PCIs identified during the I-MUR service provision, classified using the method of Krska et al.
- Patients’ adherence to asthma medication during and after the I-MUR service provision, measured using questions embedded within the I-MUR instrument.
- Cost estimated on the severity of asthma, before, during and after the I-MUR service provision.

## Discussion

This study has been developed because of the need for a new way of working for pharmacists and pharmacy in Italy. If the results are positive, the study has the potential to open up a new professional perspective for Italian community pharmacists, providing this is accepted by all stakeholders: Italian medicine control agency (AIFA = Agenzia Italiana del Farmaco), Italian Ministry of Health, Italian general practitioners and hospital doctors.

It is also important internationally, as it is one of the largest studies ever conducted in community pharmacy and the first and largest study ever conducted in Italy, involving 75% of Italian regions.

## Conclusion

This is the first trial of any community pharmacy-based pharmaceutical care intervention to be carried out in Italy. The results of this study will inform future policy and practice in Italian community pharmacy.

### Strengths and limitations

This is the first study conducted in Italy aiming to assess pharmacists’ contribution to reducing the severity of asthma; it is one of the largest studies conducted in one single country and it introduces a new systematic and quantitative research instrument: I-MUR.

The study includes only adults, does not require spirometric testing and will run for nine months. We do anticipate, given the size of the study, that problems may arise, for example, with pharmacist and patient recruitment, resulting in the study being underpowered. However we have taken account of this in our power calculation, by evaluating four possible options based on actual results from phase one. There is also the possibility of a higher drop-out rate than anticipated and, while the study will enable us to assess whether ACT score is a suitable primary outcome measure, this may prove not to be the case.

### Ethical consideration

Ethical approval was obtained from the Faculty of Science Ethics Advisory Group for Human Participants on 18th February 2014 (reference number 0281314) from the University of Kent (UK), and in Italy on June 3rd 2014 by the Ethics Committee of Spedali Civili di Brescia (Lombardia region) with reference number NP 1710, which is acting as the reference committee for all Italian participating regions.

## Abbreviations

ACT: Asthma Control Test
AIFA: Agenzia Italiana del Farmaco (the equivalent of MHRA in England)
DALYs: Disability-adjusted life years
FOFI: Federazione Ordini Farmacisti Italiani/Italian Pharmacists Federation
GINA: Global Initiative for Asthma
GP: General Practitioners
ICC: Intra cluster correlation
MRC: Medicine Research Council
MUR: Medicines use review
NICE: National Institute for Health and Care Excellence
PCI: Pharmaceutical care issue
PI: Principal Investigator
RCT: Randomised controlled trial
SPSS: Statistical package for the social science/statistical product and service solutions
T0: Time zero
T3: Time at three months
T6: Time at six months
T9: Time at nine months
